# TRAJECTORIES OF DAYS AT HOME FOLLOWING TRAUMATIC BRAIN INJURY AMONG OLDER ADULTS

**DOI:** 10.1093/geroni/igae098.2087

**Published:** 2024-12-31

**Authors:** Jennifer Albrecht, Chixiang Chen, Jason Falvey

**Affiliations:** University of Maryland School of Medicine, Baltimore, Maryland, United States; University of Maryland School of Medicine, Baltimore, Maryland, United States; University of Maryland School of Medicine, Baltimore, Maryland, United States

## Abstract

Traumatic brain injury (TBI) is a common fall-related injury among older adults that results in over 123,000 hospitalizations and 485,000 emergency department visits annually. While older adults have poorer recovery following TBI relative to younger adults, most older adults recover well from TBI. Identifying those at increased risk of poor recovery could inform appropriate management pathways, facilitate discussions about palliative care or unmet needs and permit targeted intervention to optimize quality of life or recovery. Time spent at home and not in a hospital or other health care facility each month is a patient-centered measure associated with improved outcomes. We identified unique trajectories of monthly home time following hospitalization for TBI among Medicare beneficiaries aged ≥65 using group-based trajectory modelling and identified characteristics associated with poor recovery. Among 20,350 Medicare beneficiaries who sustained TBI between 2010-2018, four unique trajectories of home time were identified: poor recovery (9.5%), improving recovery (13.7%), good recovery (66.4%), and declining recovery (10.4%). Recovery of monthly home time was complete for most by three months post injury. The strongest predictors of membership in the poor relative to the good recovery trajectory group were diagnosis of ADRD (odd ratio (OR) 2.42; 95% confidence interval (CI) 2.16, 2.72) and dual eligibility for Medicaid, a proxy for socioeconomic vulnerability (OR 5.13; 95% CI 4.59, 5.74). Future studies should seek to further characterize and investigate identified recovery groups to inform management and development of interventions to improve recovery.

